# Tertiary lymphoid structures in cancer: immune mechanisms and clinical implications

**DOI:** 10.1002/mco2.489

**Published:** 2024-03-11

**Authors:** Siyu Wang, Hua Wang, Chenbei Li, Binfeng Liu, Shasha He, Chao Tu

**Affiliations:** ^1^ Department of Orthopaedics The Second Xiangya Hospital of Central South University Changsha Hunan China; ^2^ Hunan Key Laboratory of Tumor Models and Individualized Medicine The Second Xiangya Hospital of Central South University Changsha Hunan China; ^3^ Xiangya School of Medicine Central South University Changsha Hunan China; ^4^ Department of Oncology The Second Xiangya Hospital of Central South University Changsha Hunan China; ^5^ Shenzhen Research Institute of Central South University Guangdong China; ^6^ Changsha Medical University Changsha China

**Keywords:** clinical implications, immune mechanisms, soft tissue sarcoma, tertiary lymphoid structures (TLSs), tumor immune microenvironment (TIME)

## Abstract

Cancer is a major cause of death globally, and traditional treatments often have limited efficacy and adverse effects. Immunotherapy has shown promise in various malignancies but is less effective in tumors with low immunogenicity or immunosuppressive microenvironment, especially sarcomas. Tertiary lymphoid structures (TLSs) have been associated with a favorable response to immunotherapy and improved survival in cancer patients. However, the immunological mechanisms and clinical significance of TLS in malignant tumors are not fully understood. In this review, we elucidate the composition, neogenesis, and immune characteristics of TLS in tumors, as well as the inflammatory response in cancer development. An in‐depth discussion of the unique immune characteristics of TLSs in lung cancer, breast cancer, melanoma, and soft tissue sarcomas will be presented. Additionally, the therapeutic implications of TLS, including its role as a marker of therapeutic response and prognosis, and strategies to promote TLS formation and maturation will be explored. Overall, we aim to provide a comprehensive understanding of the role of TLS in the tumor immune microenvironment and suggest potential interventions for cancer treatment.

## Graphical abstract

Tertiary lymphoid structures (TLSs) are unencapsulated aggregates of organized lymphocytes in chronic inflammatory or tumor tissues, which has been detected in a series of malignant tumors such as lung cancer, breast cancer, melanoma, virous sarcomas and so on. Previous studies have indicated that TLS could have significant clinical implications, including being a commonly favorable prognostic marker. However, these reports are usually inconsistent in different research, especially in sarcomas. Soft tissue sarcomas have extensive immunologic heterogeneity, including genetic alterations, expression of neoantigens, and transcription of immune checkpoint molecules. These immune characteristics lead to unsatisfactory immunotherapy effect and more side effects. Therefore, improved treatment strategies based on the immune characteristics of sarcoma need to be developed. The generation of tumor‐localized TLS can be facilitated by a series of therapeutic modalities, which could potentially mitigate the immunosuppressive phenotype of sarcoma and enhance treatment response. This strategy can also be extended to other malignancies as well. In this comprehensive review, we elucidate the formation mechanisms and composition of tumor‐associated TLS and provide an in‐depth discussion of immune mechanisms and clinical implications of TLS in three common cancers and soft tissue sarcomas. Combined with the existing treatment strategies, we propose possible intervention programs centered around TLS modulation, which provides a new evaluation index and treatment strategy for the treatment of cancer.

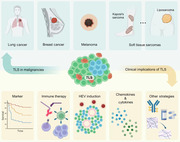

## 1 INTRODUCTION

Cancer has been the leading cause of death in China since 2010.^1^ Traditional treatments, including surgical resection, chemotherapy and radiotherapy, are restrictively available and have adverse effects meanwhile.^2^ Notably, the clinical benefit is limited to a fraction of patients.^3^ Immunotherapy has been adopted in a variety of malignancy like breast cancer (BC), lung cancer, hepatocarcinoma, and melanoma.^4^ However, some patients do not respond well to immunotherapy, especially for those with low immunogenicity, which means that the tumors are not easily recognized and targeted by the immune system.^5^ Additionally, the tumor microenvironment can be immunosuppressive, further hindering the effectiveness of immunotherapy. Sarcoma frequently exhibits both of these characteristics, rendering it a notable form of malignant tumor. To address these challenges, researchers are exploring various strategies to enhance the immunogenicity of malignancies and improve the response to immunotherapy. Currently, the expression of immune checkpoints and immune cell infiltration are the most crucial indicators of immunotherapy response in cancers,^5^ allowing for more targeted and personalized treatment approaches. However, in many cases, it does not consistently align with the clinical effect, highlighting the need to identify new markers and intervention targets.

Tertiary lymphoid structures (TLSs), also referred as ectopic lymphoid structures or tertiary lymphoid organs, is an aggregate of lymphocytes in nonlymphoid structures. Recent studies have shown that TLS is associated with a more favorable response to immune checkpoint blockade (ICB) and a higher survival rate in patients with malignant tumors.^6^ Meta‐analysis showed that TLS was associated with better prognosis and lower risk of recurrence in solid tumors.^7^ Researches specific to certain tumors such as bladder cancer and cholangiocarcinoma has also confirmed the association between TLS and favorable prognosis.^8^, ^9^ The correlation could be attributed to augmented antitumor immune responses within TLS,^10^ and promoting TLS formation has been shown to enhance immunotherapy in animal models.^11^ However, current research on the immunological mechanisms and clinical significance of TLS in malignant tumors is insufficient. Thus, we aim to summarize existing studies and propose potential future research directions.

To clarify the role of TLS, we will first outline the background of TLS and detail its composition, neogenesis, and the immune characteristics of TLS in tumors. Concerning that TLS is formed by an inflammatory reaction,^12^ and cancer is often seen as a chronic inflammatory process,^13^ we will then highlight the inflammatory response in relation to cancer development, covering both cancer‐promoting and anticancer pathways. Consequently, we will conclude by examining the immune traits of cancers and their impact on TLS characteristics. Our attention will be directed toward the role of TLSs in specific tumors such as lung cancer,^13^ BC,^14^ and melanoma.^15^ In the case of soft tissue sarcomas, we will focus on Kaposi's sarcoma (KS), undifferentiated pleomorphic sarcoma (UPS), liposarcoma, angiosarcoma and gastrointestinal stromal tumors (GISTs) for their common features including a higher rate of immunotherapy utilization and TLS detection. Additionally, we will explore the therapeutic implications of TLS, encompassing its role as a marker of therapeutic response and prognosis,^16^ as well as the promotion of TLS formation and maturation through immunotherapy,^17^ high endothelial venule (HEV) induction,^18^ cytokine and chemokine modulation,^19^ and other approaches.^20^ We anticipate that this summary will further elucidate the role of TLS in tumor immune microenvironment (TIME) and suggest a promising direction for cancer intervention.

## 2 FORMING PROCESS AND IMMUNE MECHANISMS OF TLS

Described as an aggregate of lymphocytes in nonlymphoid tissues, a clear definition of TLS is currently absent, and its maturity and cellular composition have a large variable range. Furthermore, its involvement in the local immune mechanisms of tumors also requires further elucidation. Here, we delve into the composition and maturation process of TLS, compare it with secondary lymphoid organ (SLO), and discuss the immunological characteristics of TLS in tumors.

### 2.1 Composition and structure of TLS

TLS is formed in response to chronic inflammatory stimulation such as infection,^21^ graft rejection, autoimmune disease, and malignant tumor tissues.^22^, ^23^, ^24^ Its structure closely parallels that of SLO, except that TLS lacks a capsule and afferent/efferent lymphatic vessels (LVs).^25^ HEV is a crucial structure present in both SLO and TLS, which are specialized postcapillary venules expressing L‐selectin and peripheral node addressins (PNAds) on endothelial cells (ECs).^26^ The common structure of classical TLS includes a B cell follicular clustering around the follicular dendritic cells (FDCs) and a T cell region collecting around the HEV with the presence of fibroblastic reticular cells (FRCs).^27^ CD4+ follicular helper T cells (Tfh) are the main subset of T lymphocytes, while CD8+ cytotoxic T lymphocytes (CTLs), CD4+Th1, and CD4+Tregs are also present.^6^ Stem‐like T cells have been found to predominantly localize within TLS. This suggest that TLS not only recruits lymphocytes but also provides support and protection for their differentiation.^24^ Dendritic cells (DCs) were also found in the T‐cell zone, and their abundance is correlated with elevated levels of Th and CTL infiltration, promoting B cell proliferation and antibody generation.^28^ Stromal cells include fibroblasts, FRCs, and FDCs. FDCs enhance the interaction between antigen‐presenting cells and naive lymphocytes in the B cell domain, and the FRCs, which are located in the T cell region, serve as the structural framework of TLS.^29^ The cellular composition and structure to some extent determine the functionality of TLS.

### 2.2 Cellular and molecular mechanisms of TLS neogenesis

**FIGURE 1:**
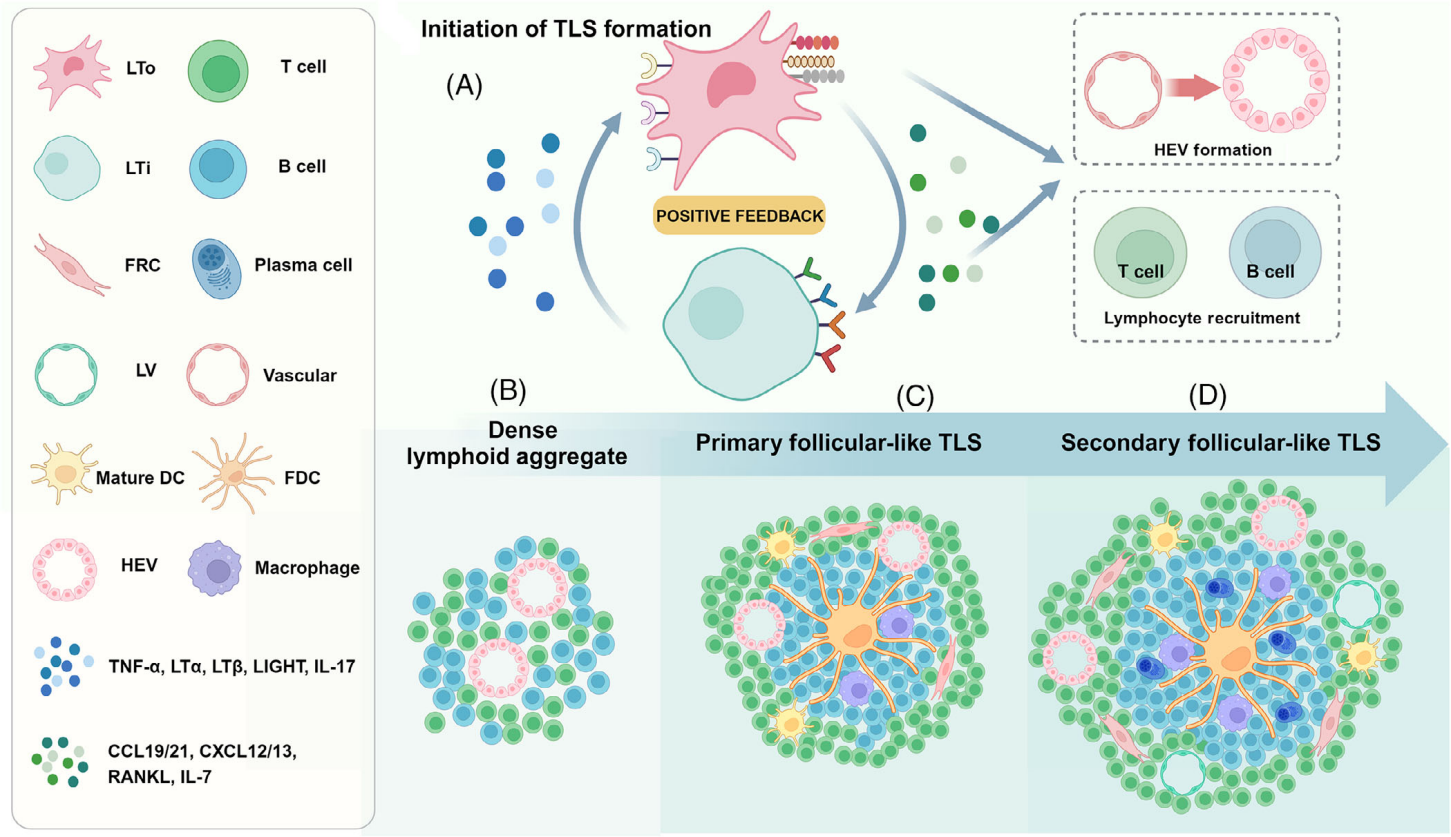
Initiation and maturation of TLS. (A) The crosstalk between LTo and LTi initiates the formation of TLS. Related factors generate positive feedback, promote HEV formation, and recruit lymphocytes. (B) Dense lymphoid aggregate without FDCs or separate B/T cell compartments. (C) Primary follicular‐like TLS has B‐cell regions surrounding FDC and T‐cell regions surrounding HEV but lacks mature germinal centers. (D) Secondary follicle‐like TLS is characterized by the appearance of mature germinal centers, which marks TLS maturation.

The mechanism behind TLS formation remains unclear, but it may be similar to that of lymph nodes (LNs), as depicted in Figure 1. The most dominant factor for TLS formation is the positive feedback formed by the interaction of lymphoid tissue organizer (LTo) cells and lymphoid tissue inducer (LTi) cells.^6^, ^30^ LTo cells, also regarded as mesenchymal precursors, comprises stromal cells, ECs, fibroblasts, FDCs, and FRCs. These can be broadly categorized as endothelium‐derived LTo and mesenchymal‐derived LTo.^31^ Hematopoietic precursors serve as LTi, including B cells, macrophages, DCs, group 3 innate lymphoid cells (ILC3), and Th17, which are mainly inflammatory myeloid cells.^32^, ^33^, ^34^ A multitude of cytokines and chemokines participate in the interaction between LTo and LTi cells, here we categorize them based on their function into three classes. The first category of factors represents upstream regulators of TLS formation, including IL‐7, LT (lymphotoxin)‐β, tumor necrosis factor (TNF)‐α, TNFSF14 (LIGHT or CD258), TNF‐α, and LT‐α. IL‐7 signal upregulates LT‐α and LT‐β and serves as the initiation of subsequent processes.^35^ LT‐β mediates the interaction between LTi and LTo cells,^30^ whereas LIGHT serves as an alternative ligand for LTβR.^36^ In adipose tissue, TNFs and IL‐α facilitate this effect.^37^ The second kind of molecules participate in the accumulation of immune cells, which include CXCL13, CCL19/21, CCL5, CCL22, CXCL12, B‐cell activating factor (BAFF), IL‐17, CCL5, CCL22, and DC lysosome‐associated membrane protein (DC‐LAMP). CXCL13 acts as the most critical factor for immune cell aggregation which initiates LN or TLS development.^30^ CCL19/21 exhibits similar functions in promoting the B/T cell compartments.^38^ Chemokines CCL5 and CCL22 play distinct roles in TLS maturation.^39^ CCL5 is involved in T cell homing, enhancing immune reactivity in tumor environment,^40^ while CCL22 primarily recruits Tregs, leading to the formation of suppressive TLS.^41^ CXCL12 produced by fibroblasts promote plasma cell survival,^42^ while TNF superfamily (TNFSF) member BAFF plays a similar role.^43^ IL‐17 primarily promotes the proliferation and secretion of FRCs,^44^, ^45^ which act as the scaffold of TLS. Another molecule of interest is DC‐LAMP, a marker of DCs. DC‐LAMP is usually associated with robust functional B/T cell infiltration.^46^ Previous research has showed that its presence is considered as a sign of TLS maturation and has been shown to inhibit LN metastasis in lung cancer.^47^ The third kind of molecules are associated with HEV formation, which affect the maturation of TLS. These are mainly adhesion molecules expressed on vascular or lymphatic endothelium, including intercellular adhesion molecule 1, vascular cell adhesion molecule 1, and mucosal addressin cell adhesion molecule‐1. These molecules participate in HEV genesis and further facilitate LTi residency.^48^, ^49^ Besides, LT‐β is also essential for HEV network generation. Mice lacking LTβR expression in EC show reduced HEV network generation and impaired lymphocyte trafficking in the LNs.^50^ In summary, these three classes of cytokines respectively mediate the initiation of TLS formation, immune cell recruitment, and HEV formation, collectively participating in the maturation process.

The development of TLSs involves three mature stages: (1) a dense aggregation of lymphocytes without separate T cell and B cell areas and FDCs; (2) primary follicle‐like TLS containing FDCs but lacking germinal center (GC) response; (3) secondary follicle‐like TLS with active GC. Central FDC network and functional GC formation are considered as indicators of maturity of TLS.^51^ The maturation and occurrence processes are illustrated together in Figure 1. Due to inconsistency in composition and maturity, different studies often employ varying definition criteria for TLS. Current methods for identifying TLS include: recognizing dense lymphoid tissue aggregates using hematoxylin–eosin stain,^52^ immunohistochemistry, immunofluorescence labeling for relevant cells, CT‐based radiomics,^53^ and gene expression profiling for chemokines.^54^ The lack of uniformity in criteria may result in variations in the significance of experimental and analytical outcomes.

### 2.3 Comparison between TLSs and SLOs

The SLOs, with LNs as representatives, bear many similarities in structure and function to TLS. However, they still have some differences. First, SLO is formed during the embryonic stage while TLS is formed after birth, and the formation of SLO is generally physiological while TLS formation is usually due to microbiota or immune response. Second, although lymphatic ECs can be found in TLS, TLS is not enveloped and does not have afferent/efferent LVs, causing consistent exposure to antigens. This unenveloped structure allows TLS to facilitate in situ adaptive immune response, thereby circumventing the need for immune cell trafficking to and from SLO.^55^ Third, LT signaling are essential for SLO formation, whereas TLS can also be activated by IL‐17 stimulation.^56^ What is more, it has been reported that TLS in adipose tissue is TNF‐dependent but not LTβR independent.^37^ Fourth, LTi may not be necessary for TLS formation and can be replaced by local immune cells, such as Th17 and ILC3.^56^, ^57^ Table 1 summarizes the differences between TLS and SLO. These characteristics reflect the more sensitive reactivity of TLS to neoantigens and its significance as a newly formed lymphoid structure.

**TABLE 1:** Comparis between TLSs and SLOs.

	TLSs	SLOs
Time of occurrence	After birth	Embryonic stage
Cause of occurrence	Microbiota or immune response	Physiological process
Envelope	−	+
Afferent/efferent LVs	−	+
Initial signal	LTs, IL‐17, TNF	LTs
Inducer cells	LTi, Th17, ILC3	LTi

### 2.4 Immune characteristics of TLS in tumors

In the majority of previous research, TLS have been identified within chronic inflammatory tissues. What is more, cancer is often recognized as a dynamic and chronic inflammatory process.^58^ Basic process involves tissue injury, cytokine production and immune cell chemotaxis. Subsequently, cells proliferate and alter the extracellular matrix in response to growth factors, thus repairing the tissue. TLS can be formed in this chronic inflammatory response, but its function is related to the characteristics of inflammatory process. Although cancer‐related inflammatory response is mostly antitumor, protumor response exists,^59^ which is mediated by indoleamine2,3‐dioxygenase1 (IDO1) activation,^60^ programmed cell death protein (PD)‐L1 upregulation,^61^ myeloid‐derived suppressor cells (MDSCs) mobilization,^62^ and T cells exhaustion.^63^ These protumor inflammatory response effects tend to occur in repressive TLS structure.

Although presence of intratumoral TLS is one of the defining features of highly immunogenic tumors,^64^, ^65^ the genetic characteristics of the tumor to some extent determine the immune response of TLS. These include Tumor mutational burden (TMB), neoantigen, immune checkpoint expression, immune cell infiltration, and so on.^66^ TMB is defined as the number of somatic mutations per coding area in a cancer genome, which is usually related to immune checkpoint expression and determines ICB response.^67^ Neoantigens, including tumor‐associated antigens and tumor‐specific antigens, are often targets of adoptive cell therapy (ACT).^68^ A representative is the cancer‐testis antigen, which is expressed in the testis, placenta, and malignant tumors but not in normal tissues.^69^ Of note, the expression levels of immune checkpoint proteins and tumor antigens within TLS determine the immune response in TLS. Common checkpoint proteins include PD‐1, CTL‐associated antigen‐4 (CTLA‐4), T cell immunoglobulin (TIM), lymphocyte‐activation gene 3 (LAG‐3).^70^ PD‐1 affects the activity of effector T cells,^71^ while CTLA‐4 affects the initial activation. Though CTLA‐4 is principally expressed on CD8+ T cells, its blockade mainly interferes with 2 T cell subsets: extends the T helper cell‐dependent immune responses, and downregulates the immune‐suppressive effects of Tregs.^71^ Checkpoint TIM and mucin domain‐containing protein (TIM) expressed on Tregs and macrophages has also received attention in recent years for its therapeutic significance.^72^ The polymorphism and expression levels of PD‐1 and TIM‐3 are highly correlated in tumor tissues, and they are jointly involved in tumor growth.^73^ The synergetic blockade of PD‐1, TIM‐1, and LAG‐3 enhances the antitumor effect of T cells while the single blockade of each is only modest.^71^ LAG‐3 encodes an inhibitory receptor similar to CD4, but it is not restricted to CD4+ T cells and is also expressed on activated CD8+T cells, natural killer (NK) cells, myeloid cells, and DCs.^74^ In addition to membrane receptors, soluble LAG‐3 exists, which impairs antitumor response in periphery blood and local tumor.^75^ These inhibitory ligands might contribute to exhausted phenotype of immune cells and repressed immune response.^76^ This also underscores the significance of modulating the immune response of TLS for tumor immunotherapy.

While TLS is widely recognized as a tumor component, its maturation and localization are influenced by various factors, which in part determine its function. The proximity of TLS to tumor cells has been shown to affect their function.^77^ Heterogeneity of cellular composition in different mature stages of TLS significantly determines its functionality. Primary follicular‐like TLS structures contain B cell aggregates, thus showcasing potential for antibody‐based therapies such as tumor vaccines. Secondary follicular‐like TLS structures feature mature T and B cell zones, making them responsive to more therapies like T cell therapies and ICB.^78^ Since mature rather than immature TLS have good immunoreactivity and prognostic value, it is of interest to clarify the influence of tumor immune characteristics on their maturity.^79^ Meylan et al.^80^ identified a subgroup of genes associated with immature TLS in hepatocellular carcinoma, mainly encoding molecules involved in immunosuppression and immune cell exhaustion. Furthermore, the differential roles of mature and immature TLS have been demonstrated in a variety of tumors. Mature TLSs are usually detected in peritumoral area and expresses signals of T cell activation, plasma cell expansion, and CXCL13 production.^81^ Single‐cell sequencing targeting BC identified mainly immunomodulatory and myeloid cell gene expression profiles associated with immature TLS.^82^ In melanoma, IKZF1 was identified as a key driver of immature TLS formation. IKZF1 did not affect melanoma cell proliferation, but its overexpression significantly inhibited TLS maturation.^83^ Intratumoral and peritumoral TLS also have distinct functions. In intrahepatic cholangiocarcinoma, colorectal cancer, hepatocellular carcinoma, BC, and clear cell renal cell carcinoma, it has been suggested that peritumoral TLS is associated with a worse survival, whereas intratumoral TLS is associated with a favorable prognosis.^9^, ^84^, ^85^, ^86^, ^87^ However, peritumoral TLS density is associated with higher maturity and anti‐PD‐1 reactivity in esophageal cancer.^88^ At present, studies on the difference between the two groups mainly focus on gene expression profiles, and intratumoral TLS is associated with lower P53 and Ki67 scores, which might explain the different location and impact.^89^ What is more, structural analysis of the tumor tissue showed that lower invasiveness, relatively intact vascular network and preserved immune response were responsible for promoting intratumoral TLS formation.^90^ Published in December 2023, a meta‐analysis focusing on digestive system tumors further confirms the association between intratumoral localization of TLS and improved survival outcomes.^91^ It is noteworthy that TLS derived from inflammation or the tumor itself may exhibit distinct functionalities, with the former creating a favorable environment for the growth of malignant cells,^92^ while tumor‐induced TLS usually contributes to antitumor immunity.^93^ The spatial distribution of immune cells within TLS also warrants investigation. A study focusing on the spatial distribution of tumor‐associated neutrophils in bladder cancer patients revealed a decrease in the density of these immunosuppressive cells with increasing distance from TLS. This phenomenon could be attributed to either a concentration gradient of chemokines or the axial diffusion of immune cells after their entry into TLS through HEV.^94^ In conclusion, the formation and function of TLS in tumors and peritumoral tissues are influenced by the genetic characteristics of the tumor and the type of inflammatory response. The composition, localization, and maturity of TLS have been demonstrated to be associated with their immune response.

## 3 ROLE OF TLS IN CANCER

TLS typically constitute integral components of the positive feedback loop in antitumor immune responses.^95^ Multiple studies have provided confirmed evidence of the significant role of TLS in specific tumor types, such as lung cancer, BC, and melanoma, which are commonly targeted by immunotherapy interventions. Low immunogenic sarcomas have also been subject to investigation in order to enhance their immunotherapeutic effects. Here, we have summarized the recent research on the role of TLS in specific types of cancer. We focus on lung cancer,^96^ BC,^86^, ^97^ and melanoma, as they are more frequently targeted for immunotherapy applications. Sarcomas are also of interest due to their resistance to conventional treatments, making immunotherapy a potential approach. TLS holds prognostic significance and serves as a potential intervention target in these tumors, thus we summarize the role of TLS on certain tumor types, and display it in Table 2.

**TABLE 2:** immune characteristics of TLSs in malignant tumors.

Tumor type	Research objects	Characteristics of TLS	Year	References
Lung cancer	Murine models and clinical samples	TLS serve as a niche for CD8+T cells to exert antitumor effect	2023	^24^
	Clinical samples	TLS maturation, B cell differentiation and IFN‐γ response can be weakened by LNs metastasis	2023	^25^
		Proliferation and secretion of CD4+T in TLS is inhibited by Tregs	2022	^101^
		Higher antigen‐presentation and T cell activation, but high CTLA4 expression in TLS	2023	^102^
		higher CD8+T and lower macrophages in TLS correlate to neoadjuvant response	2022	^106^
Breast cancer	Clinical samples	higher PD‐1, LAG‐3, and TIM‐3 in TLS(+)tumor	2022	^113^
		TLS can elicit tumor‐specific T cell clones independent of sentinel LNs	2022	^115^
		higher CD8+T cells and doubled TIL levels in TLS (+) tumor	2023	^116^
		Th1‐oriented Tfh exert antitumor effect in TLS	2021	^118^
		CD169+ macrophages, Tregs and regulatory B cells in TLS constitute inhibitory microenvironment	2023	^119^
		functional DCs mediate immune response in TLS	2023	^121^
Melanoma	Clinical samples	TLS are mainly intratumoral than peritumoral with higher B cell proliferation in primary tumor	2023	^125^
		TFR exert immunosuppressive effect in TLS	2021	^127^
		AID (+) B cells relate to positive prognosis and CD21 (+) B cells relate to negative prognosis	2021	^128^
		TLS formation is accompanied by nave T cell recruitment	2022	^131^
KS	Murine model	Upregulated Th2 response and alternated activation of macrophages	2022	^136^
UPS	Clinical samples	TAMs are predominant and T cells are diffused in TLS	2020	^144^
		High OX‐40 on TLS‐related Tregs inhibited T cells proliferation	2015	^153^
Liposarcoma	Clinical samples	TLS act as the only antigen‐presentation site with colocalization of DCs and T cells	2015	^156^
		High CD8+ T cells, Tregs, and B cells in TLS	2019	^157^
Angiosarcoma	Clinical samples	PD‐1 is detected only on T cells that contact B cells in TLS	2021	^159^
GISTs	Clinical samples	More B cells and fewer Tregs in TLS (+) tumor	2020	^162^
		Macrophage infiltration and IDO1 expression leads to immunosuppressive TLS	2018	^164^

Abbreviations: GIST, gastrointestinal stromal tumors; IFN, interferon; TAM, tumor‐associated macrophage.

### 3.1 TLS‐related immune responses in lung cancer

Lung cancer has gain wide attention for its high occurrence and mortality.^98^ Although immune therapy has changed the treatment paradigm, insensitivity of some individuals has confined its appliance. Therefore, several researchers have paid attention to the heterogeneity and clinical implications of TLS.

In non‐small cell lung cancer (NSCLC), high density of peritumoral TLS and intratumoral TLS are associated with early stage and better postoperative survival.^99^, ^100^ Tregs is commonly seen as the immunosuppressive component in TIME,^98^ Devi‐Marulkar et al.^101^ reported that the Tregs in NSCLC‐associated TLS inhibits the proliferation and cytokine secretion of CD4+ T cells, which causes poor clinical outcome. However, antibodies against CTLA4 and glucocorticoid‐induced TNF receptor‐related protein lift this inhibition, which suggests that Treg function is the key point of antitumor immunity in NSCLC‐related TLS.^101^ CD8+T cells in lung cancer have been reported to preferentially localize in TLSs rather than tumor parenchyma, which provide a protective niche for CD8+T to exert antitumor effect.^24^ Spatial expression profiling of NSCLC demonstrated significantly high levels of antigen presentation and T‐cell activation in TLS. However, its high expression of CTLA‐4 may lead to immune dysfunction.^102^ Another analysis of the correlation between clinicopathological and transcriptomic features and prognosis of lung adenocarcinoma reached a similar conclusion.^103^ In addition to local cellular mechanisms, TLS also interacts with tumor metastasis. He et al.^25^ recently reported that tumor‐derived LNs metastasis reduced TLS maturation and GC formation and was associated with poor prognosis, diminished B cell differentiation and weakened interferon (IFN)‐γ response. Therefore, mature TLS is proven to prevent LN metastasis^47^ Therefore, computerized TLS density have been used to evaluate prognosis of lung adenocarcinoma.^104^

Neoadjuvant chemotherapy has achieved good effect in NSCLC, while the intrinsic mechanism has remained unclear. A phase 2 NeoCOAST Platform Trail reported that markers of TLS maturation are associated with major pathological response of NSCLC patients treated with neoadjuvant.^105^ Another study revealed that neoadjuvant chemotherapy increased the number and maturity of TLS compared with conventional chemotherapy, and the latter can be used as an independent prognostic factor for disease‐free survival. Meanwhile, patients who achieved a major pathological response had higher CD8+T cells and lower M1 or M2 macrophages in TLS.^106^ In addition, neoadjuvant chemotherapy responders tend to have higher percentages of CD3+, FOXP3+, and CD8+/PD‐1+ cells, thus the cellular composition profile of TLS can be used to predict those patients who will benefit from neoadjuvant chemotherapy.^107^ Interestingly, although IDO1 expression is commonly associated with immunosuppression and tumor invasion, IDO1 is associated with improved therapeutic response in NSCLC treated with PD‐L1 blockade,^108^ which is not observed in conventional chemotherapy.

### 3.2 TLS in BC

BC has received extensive attention due to its high incidence. However, due to the low immunogenicity, the results of immune checkpoint inhibition therapy are not ideal.^109^ In 2021, Zhao et al.^7^ reported that TLS was associated with lower grade, stage and smaller tumor volume of BC through meta‐analysis. Furthermore, some researchers have used radiomics to reveal that TLS+ BC patients have higher long‐term control rate and better prognosis.^110^ TLS‐associated gene signature is also a promising biomarker for BC TIME and prognosis.^111^

Although the presence of TLS reduced BC invasion and metastasis, this correlation was heterogeneous between different molecular subtypes.^112^ A cross‐sectional study showed that TLS was most frequently observed in the HR (−) /HER2 (+) subtype and in the base‐like subtype.^113^ In addition, Bertucci et al.^114^ reported that in triple‐negative BC treated with neoadjuvant chemotherapy, the high TLS score group had a significantly higher pathological complete response. The investigators further found that receptors for the immune checkpoint molecules PD‐1, LAG‐3, and TIM‐3 were more common in tumors containing TLS and with high tumor‐infiltrating lymphocyte (TIL) density, which might explain its prognostic significance.^113^ Single‐cell immune profiling revealed that TLS can elicit tumor‐specific T cell clones independent of sentinel LNs in primary breast tumors.^115^ Compared with those devoid of TLS, BC with TLS had a higher proportion of CD8+T cells in the tumor core and doubled TIL levels.^116^ However, the cellular subset function of TLS in BC is highly heterogeneous. Occurrence of CXCL13‐producing Tfh is the sign of TLS maturation. Functional analysis showed that only Th1‐oriented Tfh cells exert adaptive immunity and that this subset is regulated by T follicular regulatory cells (Tfr).^117^ Maintenance of the balance between Tfh and Tfr is a prerequisite for the antitumor effect of TLS.^118^ CD169+ macrophages at draining LNs are considered to be favorable prognostic factors. However, in the primary lesion, CD169+ macrophages are often located within TLS, which is rich of Tregs and regulatory B cells. This immunosuppressive microenvironment makes this macrophage subtype have poor prognostic significance in primary tumor.^119^ Using single‐cell transcriptome sequencing, Wang et al.^120^ identified CD23 as a biomarker of TLS in BC and proposed that heterogeneity of B cells in TLS determines the efficacy of immunotherapy. DC heterogeneity has also been of concern, and investigators determined that the functional status of CCL19‐expressing DCs correlated with favorable anti‐PD1 responses, T‐cell immunity, and the presence of TLS.^121^ Other immune cell markers in TLS tissue of BC, including FoxP3, CD4/CD8 ratio, and CD68, are reported to be independent factors for poorer survival, thus suggesting its pathological significance.^122^

### 3.3 TLS in melanoma

TLS has been subject to more comprehensive research in melanoma due to its frequent detection in this tumor, with potential implications for other types of cancer. Some lymphocyte aggregates in melanoma identified as TLSs are often accompanied by PD‐L1 expression.^123^ In 2022, Li et al.^124^ reported that the 12‐chemokine TLS signature score reflected higher tumor immune status and had predictive and prognostic value. Compared with metastatic melanoma, TLS in primary melanoma is mainly located intratumor rather than peritumoral, with higher B cell density and proliferation fraction, and therefore more immunoactive.^125^ A series of studies have investigated the structure and components of TLS in melanoma, including HEV, fibroblasts, and B cells. In cutaneous melanoma, HEV is mainly found within TLS structures and its density is strongly correlated with the density of T and B cells.^126^ Of interest, Eschweiler et al.^127^ pointed out that a new cell subset located primarily in TLS, follicular regulatory T cells (Tfr), exerts a stronger suppressive effect than Tregs. Furthermore, anti‐CTLA‐4 therapy to deplete Tfr cells followed by anti‐PD‐1 therapy strengthens ICB efficacy, thus resulting in better clinical outcomes.^127^ Rodriguez et al. highlighted the importance of cancer‐associated fibroblasts in the formation of TLS. Markers of B‐cell function in TLS have also been shown to have prognostic value, with AID (+) B cells showing a positive correlation and CD21 (+) B cells showing a negative correlation.^128^ CXCL13 mediates the recruitment of B cells and releases LTα1β2 as an upstream mechanism to promote the recruitment of fibroblasts and activation of IFN signaling, thereby inducing TLS formation.^129^, ^130^ Hoch et al.^131^ analyzed immune infiltration in metastatic melanoma and proposed that TLS formation may also be accompanied by the recruitment of naïve and naïve‐like T cells that can predict the response to ICB. Genes driving TLS formation in melanoma have also received attention. Yin et al.^83^ found that the IKZF gene, although it did not affect the proliferation of melanoma cells, led to the formation of immature TLS, resulting in a greater tumor burden.

### 3.4 TLS‐related immune responses in sarcomas

Sarcoma is a distinct type of malignant tumor derived from mesenchymal tissue.^132^ It be broadly categorized into those of osseous and soft tissue origin., accounting for 20 and 80%, respectively.^133^ Most sarcomas exhibit poor response to traditional treatment regimens, and the effectiveness of immunotherapy is often compromised due to low immunogenicity and the concurrent presence of an immunosuppressive microenvironment in sarcomas. Thus elucidating the immune mechanisms of TLS and applying them in clinical settings holds promise for improving the clinical management of sarcoma patients. Here we focus on KS, UPS, liposarcoma, angiosarcoma, and GISTs, for their relatively high incidence. Research focusing on them has implications for other subtypes of sarcomas.

#### 3.4.1 TLS in KS

KS is defined as an angio‐proliferative disease specially induced by human herpes virus 8, which is also known as KS‐associated herpesvirus (KSHV). It usually presents as pink or purple painless plaque or nodules of the mucocutaneous skin that do not fade with pressure, common locations also involve LNs and visceral organs.^134^ Viral infection and immune impairment or dysfunction are initiating factors of oncogenesis. KSHV interferes with the antiviral response of host cells and regulates the expansion, activation, and phenotypic transformation of immune cells in the infected location, it also resides in B cells and macrophages during latency.^135^ Ectopic lymphoid structures are often formed in KSHV infection, which promotes the activation of T and B cells and the generation of germinal centers. On the one hand, viruses may lurk in such structures and cause delayed infection; On the other hand, ectopic lymphoid structures are to some extent involved in antisarcoma immunity.

Virus‐induced mice model has been established to study the progression of KS in humans. Gangadharan et al.^136^ detected upregulated Th2 response and alternative activation of macrophages in KS mice model, which is secondary to sarcoma and may affect immune cell phenotypes and viral latency. Bronchus‐associated lymphoid tissue (BALT), which is classified as an ectopic lymphoid structure, has been detected to harbor the virus during latency, which might associate with the malignant transformation.^137^ Kocks et al.^138^ confirmed that viruses are resident in BALT structures containing abundant B cells. Concurrently, microRNAs derived from KSHV can promote germinal center reactions by reprogramming B cells.^139^ KSHV‐derived cyclin can work as the activator of cyclin‐dependent kinase 6 and regulates a series of cellular processes, especially the initiation of lymphoma.^140^ The noncanonical NF‐kB pathway is implicated in GC responses and viral persistence. Inhibiting this signaling pathway prevents latent infection.^141^ The above study investigated the involvement of ectopic lymphoid structures in the pathogenesis and immune response of KS. It also indicated the potential involvement of TLSs in the immune response.

#### 3.4.2 TLS in HMF/UPS

**FIGURE 2:**
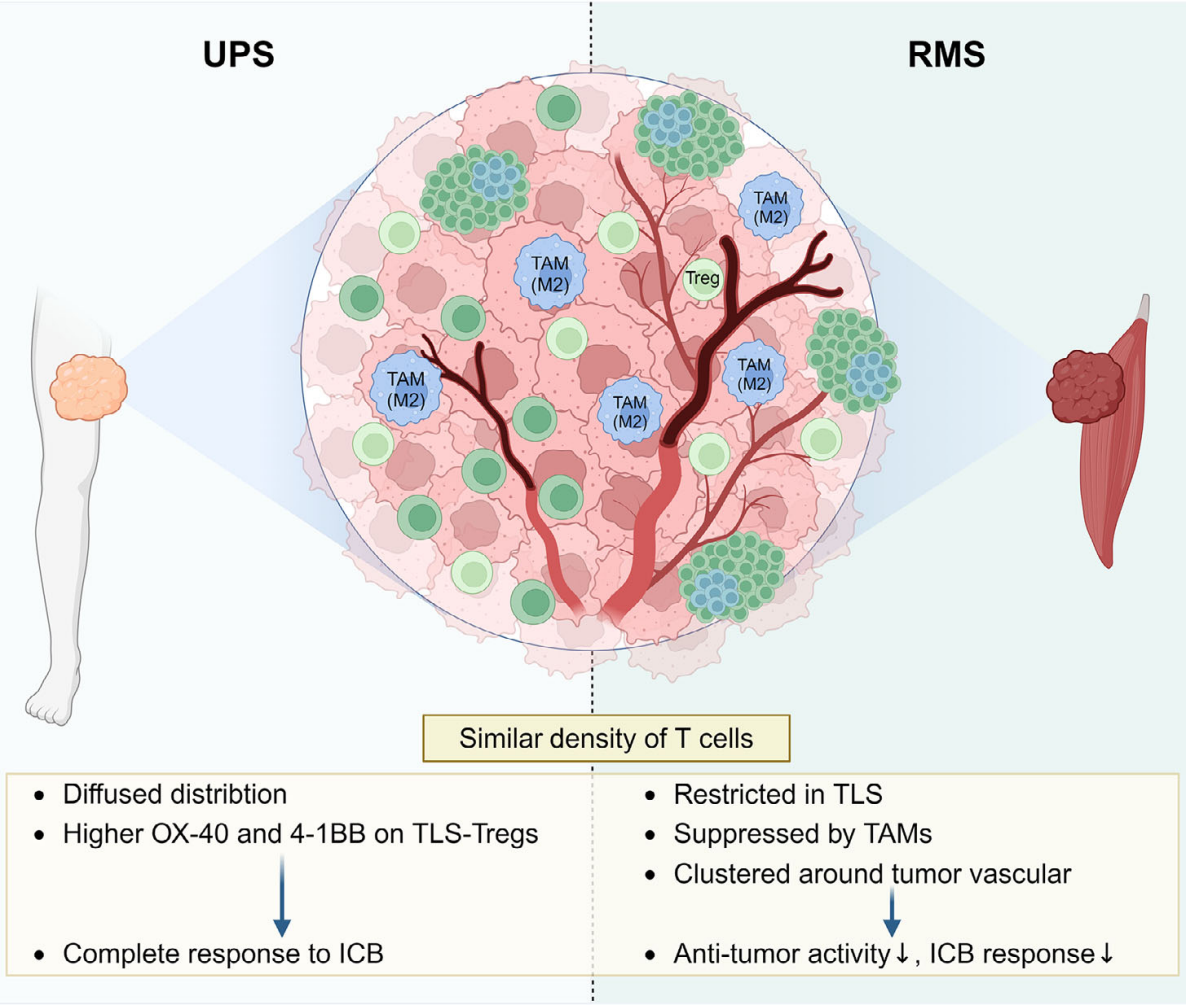
Comparison of TLS in UPS and RMS. The difference in immune infiltration between UPS and RMS might determine their ICB response. Though their T cell densities are similar, UPS generates fewer TLS structures, thus its T cells are more diffused. TLS‐Tregs express high OX‐40 and 4‐1BB, which comes to targets for ICBs. These contribute to a complete response to ICB. T cells in RMS are restricted in TLS and suppressed by TAMs. They have been observed to cluster around abnormal tumor vascular. All the above results in depressed antitumor activity and ICB response.

As the most prevalent type of sarcoma in adults, UPS is representative of a genitally complex type of sarcoma.^142^ Characteristically, the UPS has a complete response to ICB,^143^ which might be associated with the genetic factor and the local environment. Chen et al.^144^ compared the immune cell infiltration of UPS and rhabdomyosarcoma (RMS), which showed contrast responsiveness to ICB. They found that though T cell densities are similar, their distribution differs. T cells are diffuse in tumor tissue and fewer TLSs are found in UPS cases. While in RMS, T cells clusters with B cells to form TLS, and they are restricted in TLS and not elsewhere.^144^ Chen et al.^144^ also confirmed that tumor‐associated macrophages (TAMs), mainly the M2 subtype, dominate the immunosuppressive microenvironment of UPS and RMS, and most TILs are proximate to the tumor vasculature in both. Therefore, they suggested that since the RMS T cells are restricted in TLS and inhibited by immunosuppressive cells, their cytotoxic ability is depressed. The comparison between TLSs in UPS and RMS is depicted in Figure 2. To elucidate the cause of this immunosuppressive environment, they detected significantly higher expression of colony‐stimulating factor (CSF)‐1 receptor in RMS tissues compared to UPS. CSF1R promotes the survival and differentiation of immunosuppressive macrophages, maintaining an anti‐inflammatory environment to promote tumor growth.^145^ The levels of TAMs, particularly the M2‐polarized subtypes, were found to have a negative correlation with B and T cell levels.^146^ This suggests that TAM is a major contributor to intratumoral TLS as an immunosuppressive niche.^144^ Recruitment of TAMs is commonly initiated by IL‐4/13 released by damaged cells in tumor tissue.^147^ These cytokines activate the JAK–STAT pathway of macrophages to turn on anti‐inflammatory genes including arginase and resistin‐like molecule α, thus effectively facilitate the recruitment of immunoregulatory macrophages.^148^, ^149^, ^150^ In 2022, Wang et al.^151^ uncovered that STAT3/CCL2 axis also participate in TAMs recruitment. Whether these pathways are involved in TAM recruitment in UPS and RMS remains to be tested. Since they also reported that most TILs are proximate to the tumor vasculature, normalization of tumor vascular might accelerate the transportation of T cells. Therapies to eliminate suppressor cells can also be combined with ICB to strengthen its effect.

As costimulatory molecules, OX40 and 4‐1BB are correlated with the cytotoxicity and survival of T cells.^152^ Melake et al.^17^ estimated OX40 and 4‐1BB in a retrospective cohort of UPS and liposarcoma patients treated with radiotherapy and immunotherapy. They identified that OX40 expression on the TLS‐associated Tregs are significantly higher than the diffused Tregs, and the depletion of this phenotype upregulates CD4+ and CD8+ T cell proliferation.^153^ Thus, anti‐OX40 therapy might result in tumor site‐specific Tregs depletion and enhance the efficacy of ICB. However, since OX40 and 4‐1BB expression levels on Tregs are related to cellular localization, TLS‐related and TLS‐independent Tregs respond differently to treatment targeting OX40 and 4‐1BB. Thus, Melake et al.^17^ provided a new patient stratification metric according to the relation of Tregs to TLS to choose appropriate treatment strategies.

#### 3.4.3 TLS‐related immune responses in liposarcoma

**FIGURE 3:**
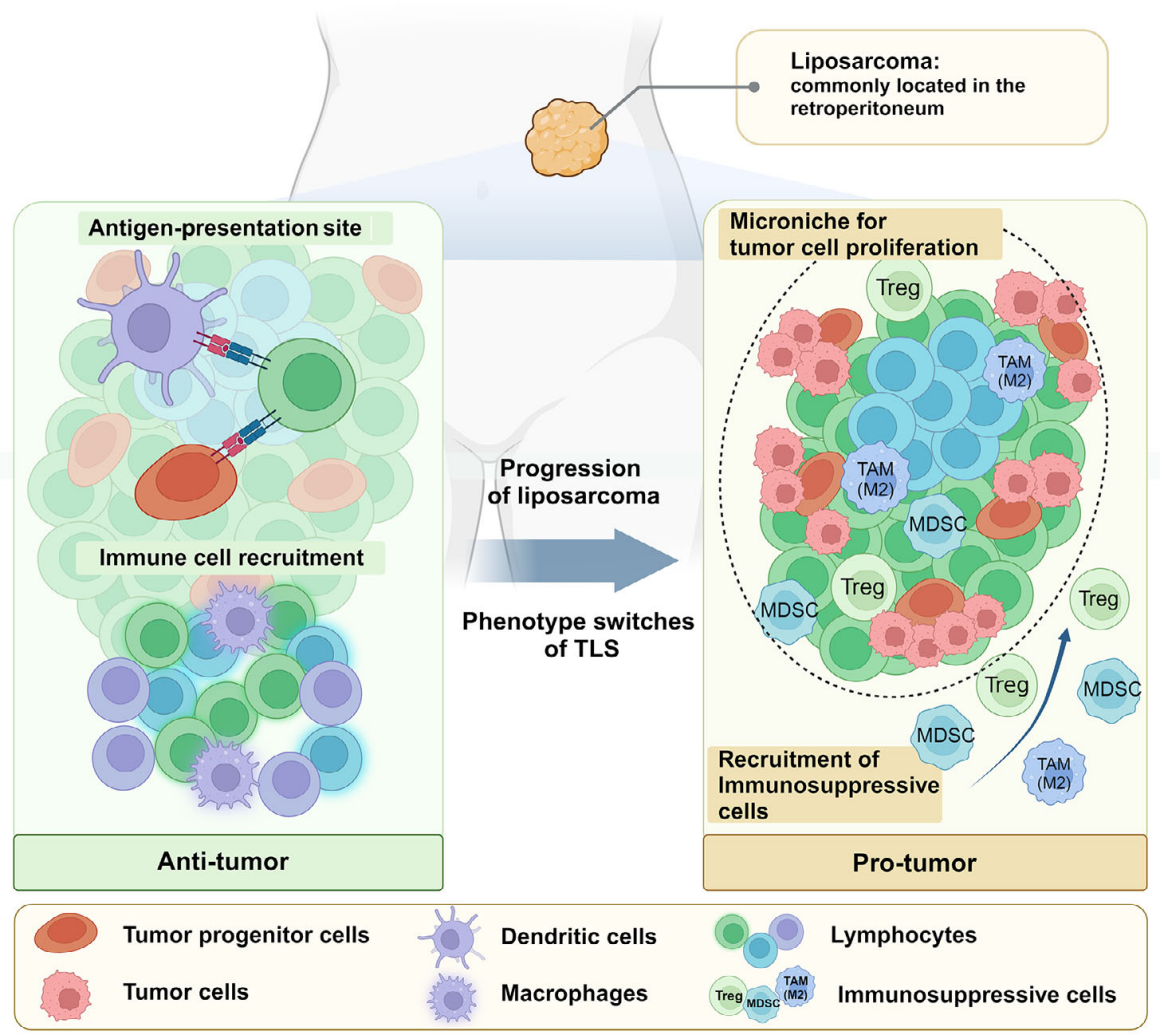
Dual role of TLS in liposarcoma. Liposarcoma commonly occurs in the peritoneum. With the progression of liposarcoma, TLS switches from antitumor phenotype to protumor phenotype. At the early stage, TLS serves as the antigen‐presentation site between dendritic cells/tumor progenitor cells and T cells. It also recruits immune cells to enhance antitumor immunity. During late stages, TLS might act as a microniche that prevents tumor progenitor cells from being attacked and recruiting immunosuppressive cells. Thus, TLS contributes to tumor cell proliferation.

Liposarcoma is an adipocytic‐origin malignancy that is commonly located in the retroperitoneum, which can be divided into well‐differentiated/dedifferentiated (WD/DD) liposarcoma.^154^ Structural and genomic analyses have revealed that liposarcoma has subtype‐specific key mutations in addition to some common mutations and translocations such as the frequently mutated gene TP53. However, some subtypes like DD liposarcoma do not have characteristic mutations or fusion genes,^155^ which may limit the use of targeted therapy. Tseng et al.^156^ confirmed the presence of TLS in perivascular and adipocytic areas in liposarcoma, which mainly consists of CD4+ T cells and a small proportion of CD8+ T cells. They observed DC‐LAMP‐positive mature DC next to CD4+ T cells, which suggests TLS as a site of antigen presentation.^156^ But surprisingly, their clinicopathologic statistics indicate that TLS was surprisingly associated with worse clinical outcomes in both WD/DD liposarcoma patients. Since liposarcoma doesn't disseminate to regional LNs, they hypothesized that TLS might be the only antigen‐presentation site, which evolves from antitumor phenotype to protumor subtype during tumor growth. Thus, the antigen‐presentation mechanism in TLS is different from solid tumors at cellular and cytokine levels.^156^ Another reason may be the inactivation of intratumoral CD8+T cells. T cells in TLS of liposarcoma can be normally expanded and sensitive to tumor antigens. However, T cells in a nonactivated state with high expression of PD‐1 would cause the downstream immune deficiency.^156^ Taken together, we propose that TLS, as a localized immune‐cell aggregate, switches from antitumor to protumor during liposarcoma development. Specifically, they range from facilitating local antigen presentation and immune cell recruitment to recruiting immunosuppressive cells to provide immune escape compartments. Figure 3 illustrates the process of this phenotypic switch. Another study reported a different prognostic effect of TLS. Yan et al.^157^ analyzed the immune characterization of 4 subtypes of retroperitoneal liposarcoma and detected TLS structure or immature TLS in WD/DD liposarcoma and myxoid cell liposarcoma, but not pleomorphic liposarcoma. Prescence of TLS is correlated with higher CD8+ T cells, B cells, and Tregs. Thus, the researchers concluded that TLS in liposarcoma is a characteristic of immune activity and longer disease‐free survival. They also revealed that tumor progression is accompanied by decreasing in TILs and increasing in PD‐(L)1 expression. To explain the inconsistency with previous research, they emphasized that the immune status of sarcomas varies according to tumor grade, subtype, and progression, as well as individual heterogeneity.^157^ In conclusion, as an antigen‐presenting site in liposarcoma, the immunological effect of TLS is closely related to its cellular composition and function.

#### 3.4.4 TLS‐related immune responses in angiosarcoma

Angiosarcoma is an aggressive vascular neoplasm with uncertain efficacy with ICB,^158^ thus some researchers tried to focus on its immune traits and its correlation with treatment. Magara et al.^159^ estimated PD‐1 and TLSs in cutaneous angiosarcoma specimens to study the correlation with prognosis. Like the results in other sarcomas, the presence of TLS was shown to correlate with higher disease‐specific survival rates in both primary and recurrent lesions. They also found that the number of TLSs negatively correlated with the differential level of tumors.^159^ Another study found that PD‐1 was detected only on T cells that were in contact with B cells, but not on surrounding T cells, suggesting that the local interaction with B cells may be related to the expression of PD‐1 on T cells, which in turn affects the efficacy of ICB.^159^ However, a retrospective study in 2022 showed no correlation between PD‐L1 level, lymphocytes infiltration and ICB response. At the same time, they found that the levels of NK cells, cytotoxic T cells, and DCs were associated with higher progression‐free survival, while tumor‐associated fibroblasts present an opposite scenario.^158^ Taken together, the previous researches showed that the function of TLS is not only related to cellular composition, but also affected by the localization of specific functional immune cells in angiosarcoma.

#### 3.4.5 TLS‐related immune responses in GISTs

GISTs, generally driven by mutations of KIT or platelet‐derived growth factor receptor α, have relatively low mutation burden. Although studies have shown that the degree of immune cell infiltration is not low, especially in TLS region, immunosuppressive cells might limit its immune response. TAMs, especially M2‐polarized macrophages take up the majority and exert protumor effects.^160^ The second common groups are T cells, among them CD8+ T cells are especially highly infiltrated.^161^ B cells expressing CXCL13 have also been highlighted for their role in TLS. Lin et al.^162^ investigated the postoperative outcomes of GISTs patients and found TLS in 44.9% of the patients. They identified TLS as an independent prognostic factor, since their association with a better differentiation state, higher overall survival, and lower recurrence rate.^162^ They also observed lower B cells and higher Treg numbers in patients without TLS, suggesting that TLSs contribute to local antitumor immunity.^162^ The above findings suggest the significance of regulating the degree of infiltration and polarization of immune cells. Since IDO1 has the potential to inhibit CD8+ T cells, upregulate Tregs and polarize macrophages into immunotolerant phenotype,^163^ IDO1 targeting might show its influence on TLS composition and the function. A Clinical Trial published in *JAMA Oncology* in 2018 showed that PD‐1 treatment of soft tissue sarcomas, especially GISTs, resulted in an immunosuppressive microenvironment characterized by macrophage infiltration and high expression of IDO1.^164^ Therefore, altering the suppressive microenvironment within the tumor, particularly in the TLS, and modulating its cellular composition, can enhance its immunoreactivity.

## 4 CLINICAL IMPLICATION OF TLS IN MALIGNANT TUMORS

**FIGURE 4:**
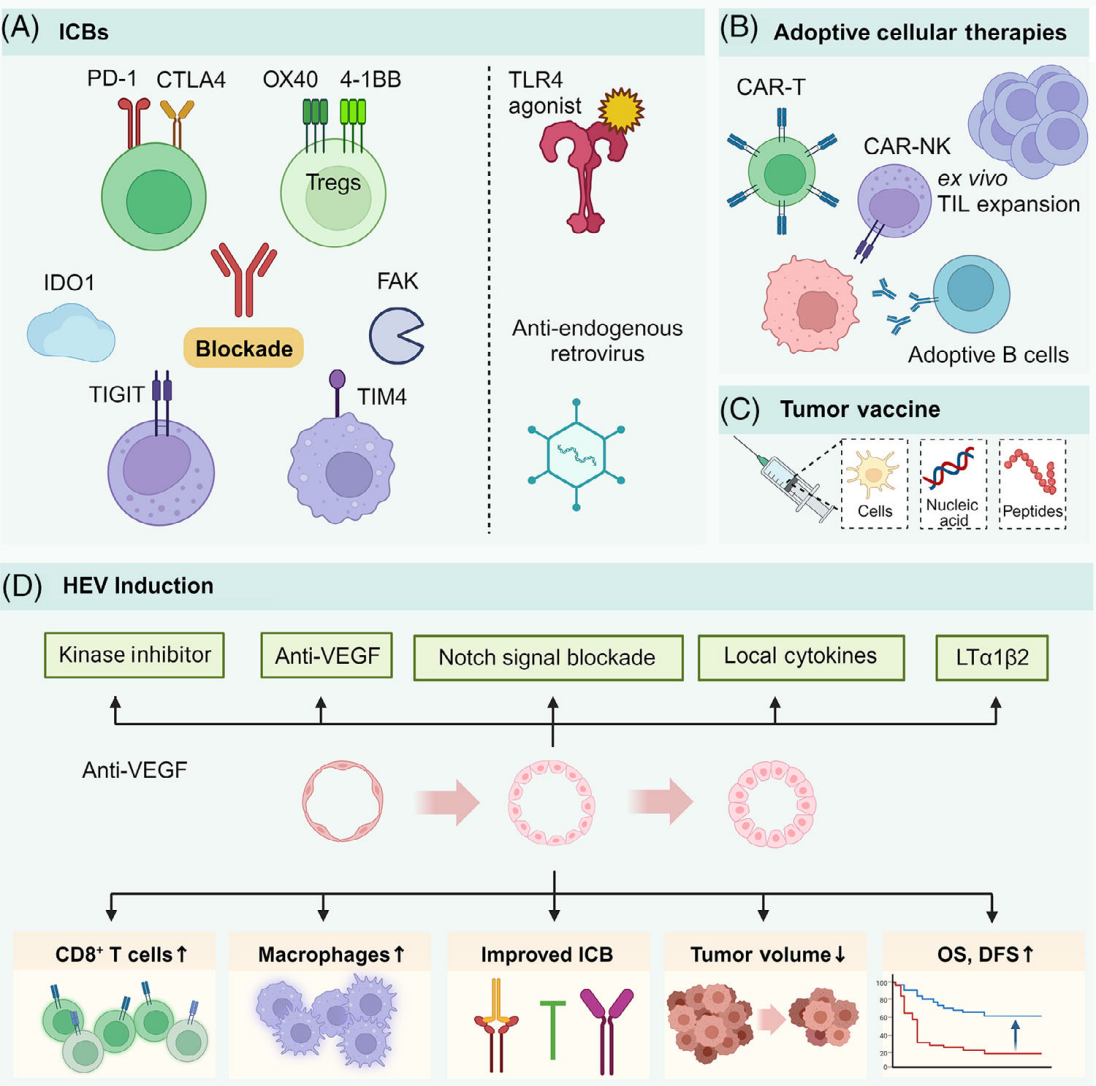
Main strategies to promote TLS formation and maturation. (A) The targets of ICB can be diverse, encompassing PD‐1 and CTLA‐4 on T cells, OX40 and 4‐1BB on Tregs, TIGIT on NK cells, and TIM4 on macrophages. Additionally, targeting enzymes such as IDO1 and FAK, applying TLR4 agonist or antiendogenous retrovirus therapy can also enhance the efficacy of ICB. (B) Adoptive cell therapies include ex vivo expansion of TIL, CAR‐T, CAR‐NK, and adoptive transfer B cells. (C) Tumor vaccine includes cell vaccine, nucleic acid vaccines, and peptide vaccine. (D) Kinase inhibitors, anti‐VEGF, Notch signal blocking, local cytokine injection, and continuous stimulation of LTα1β2 promotes HEV formation. HEV improves CD8^+^T and macrophage infiltration and ICB reactivity, which correlates with reduced tumor volume and prolonged survival.

**FIGURE 5:**
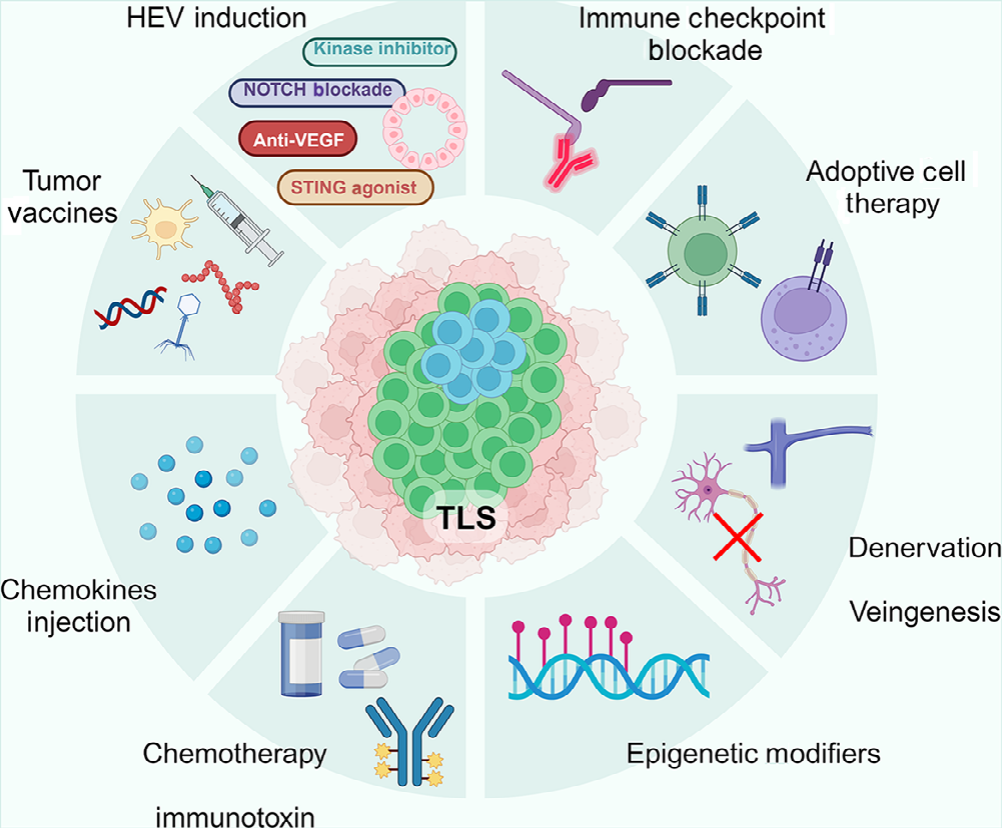
Therapies related to TLS function. Several therapeutic strategies modulate TLS function. Immune therapy, including ICB, ACT, and tumor vaccine, might activate TLS and improve local immune infiltration. Kinase inhibitors, notch blockade, anti‐VEGF and STING agonist induces HEV generation. Chemotherapy, local injection of chemokines, and immunotoxin also induce TLS formation. Recent research advancements in TLS formation include epigenetic modifications, denervation, and promotion of venous development, which could be potential areas of treatment.

Concerning the previous description, cancers with high heterogeneity can render traditional treatment ineffective in some cases due to the diverse immune characteristics of malignant tumors. Developing new therapeutic strategies based on these immune characteristics is a feasible approach.^165^ While immunotherapy has been integrated into the treatment of carcinomas and sarcomas, the response to it varies significantly.^143^, ^164^, ^166^ Research across different tumors has demonstrated that TLSs can serve as both a prognostic marker and an intervention target. Building on previous studies, we have explored methods and roles in inducing TLS formation and maturation in cancer therapy. Major strategies are illustrated in Figure 4 and Table 3. Besides, a wide range of therapies contribute to modulating TLS function, which we have summarized in Figure 5.

**TABLE 3:** Current treatment strategies associated with TLS regulation.

Treatment strategy associated with TLS		Effect of therapy	Disease	Year	References
ICB	Inhibition of OX40 or 4‐1BB	Depletion of Tregs and upregulation of CD4+T and CD8+T cells	UPS and liposarcoma	2022	^17^
	TIGIT blockade	Restore NK cell activity and promote TLS formation	Ovarian cancer	2022	^177^
	Anti‐TIM4 in non‐TLS area	TIM4+MΦ in non‐TLS area leads to immunosuppression	Various cancer types	2022	^72^
	TLR4 agonist combined with ICB	Promoted lymphocytes infiltration	Soft tissue sarcomas	2022	^180^
	PI3K blockade combined with ICB	Depletion of Tregs and upregulation of CD8+T cells	Prostate cancer	2022	^181^
	Coinhibition of PD‐1 and CTLA‐4	Enhanced ICB efficacy	Soft tissue sarcomas	2022	^16^
	IDO1 blockade	Upregulate CD8+T cells and polarize MΦ to antitumor phenotype	Soft tissue sarcomas	2018	^164^
	Coinhibition of PD‐1 and IDO1	Enhanced ICB efficacy	Lung cancer	2023	^108^
	Antibodies against endogenous retroviruses	Enhance B cell response	lung cancer	2023	^182^
ACT	TIL ex vivo expansion	Proliferation of immune active lymphocytes	Pancreatic cancer	2016	^186^
	CAR‐NK	Induce tumor cell lysis, avoid of GvHD	Melanoma	2021	^189^
	IgG1‐mutated B cells	Enhanced BCR signaling and antitumor immunity	Colorectal cancer	2022	^191^
Tumor vaccine	Protein vaccine combined with adjuvant	Promote TLS formation	Melanoma	2021	^196^
	DC vaccine combined with dasatinib	Promote TLS formation	Melanoma	2021	^197^
	TLR3 agonist injection	Enhanced tumor‐specific T cell expansion	Melanoma	2022	^198^
HEV induction	Coinhibition of VEGFR2 and PD‐1	Enhance HEV formation and CTL infiltration	Pancreatic cancer, BC, glioblastoma	2017	^207^
	Combination of a multikinase inhibitor and PD‐L1 blockade	Enhanced responsiveness in ICB‐resistant patients	19 types of progressive cancer	2023	^208^
	Combination of LTα1β2 and anti‐VEGF	Enhance HEV formation and TLS maturation	BC	2022	^18^
	Notch signaling blockade	Arterial endothelium converse to HEV phenotype, TLS formation	Chronic inflammation	2022	^211^
Cytokines and chemokines modulation	Combination of ASPH‐targeted λ phage vaccine and PD‐1 blockade	Contributes to TLS formation and promotes the cytotoxic effect of CTL	BC and Hepatic cancer	2022	^217^
	STING Agonist	Upregulate antiangiogenic factors and TLS‐inducing factors	Melanoma	2021	^19^
	Oncolytic adenovirus encoding IL‐15	Activate STING pathway to induce TLS and suppress tumor	Melanoma	2022	^221^
	gp130/STAT3 axis activation	Upregulate CXCL13, CCL19/21, promote TLS genesis	Gastric cancer	2018	^222^
	Upregulation of LTs	CCL19 and CXCL13 upregulation, lymphatic network formation in TLS	Lung cancer	2023	^223^
	Upregulation of IL‐7	Initiation of LVs genesis in TLS	Chronic inflammation	2022	^224^
	CXCL13/CCL21 local injection	Enhance B cell‐mediated DC activation, promote TLS formation	Chronic inflammation	2016	^226^
Other ways for TLS maturation	Immunotoxin LMB100	promote TLS formation	Mesothelioma	2022	^20^
	Epigenetic modifier	Maturation of immunofibroblasts and TLS stromal	Autoimmune diseases	2023	^228^
	Denervation	Relieve the inhibition of the formation of TLS by sensory nerves	Melanoma	2022	^229^
Side effect prevention	Downregulation of IL‐21/CXCL13 in PD‐1 blockade	Downregulate circulating CXCL13 and IgG deposition	Melanoma	2022	^231^

Abbreviations: CAR, chimeric antigen receptor; GvHD, chimeric antigen receptor; PI3K, chimeric antigen receptor; STING, stimulator of interferon genes; TIGIT, stimulator of interferon genes; TLR, stimulator of interferon genes; VEGF, vascular endothelial growth factor.

### 4.1 TLS as a prognostic marker

Prior research revealed that TLSs are frequently found in carcinomas and sarcomas, particularly in certain types such as lung cancer, melanoma, liposarcoma and leiomyosarcoma.^64^ Given the role that TLS plays in the TIME, its presence can often serve as an indicator of immunotherapy response. Furthermore, the presence or absence of TLS can be used to stratify patients for different treatment approaches. Tamiya et al.^167^ reported that while PD‐L1 expression and TMB did not differ between TLS+ and TLS‐ patients with lung adenocarcinoma, TLS independently correlated with better survival. However, in DD liposarcoma, TLS is reported to be protumor and associated with a worse prognosis in some research, suggesting that the role of TLS is heterogeneous.^156^ In 2022, A multicohort phase 2 study of pembrolizumab combined with low‐dose cyclophosphamide in patients was published in *Nature Communication* with advanced soft tissue sarcoma. Results showed that patients with TLS structures had higher intratumoral plasma cell abundance, 6‐month non progression rate, and objective regression rate.^16^ A recent prospective study on PD‐1 blockade patient cohort investigated the association between B cell enrichment within the GC of TLS and prolonged survival.^168^ TLS also exhibits prognostic implications independent of ICB. For patients with surgically resected melanoma, TLS can serve as a predictor of recurrence rate and survival outcomes.^128^ Similar evidences have been obtained from phase II clinical trials investigating CDK6 inhibition as a treatment for BC.^169^ Furthermore, recent studies have provided prospective evidence supporting the correlation between the presence of TLS structures and a reduced recurrence rate in squamous cell carcinoma.^170^ However, due to diverse functions of intratumoral and peritumoral TLS, it is imperative to evaluate them separately. Scoring systems specific to these 2 types have been applied in hepatocellular carcinoma to predict prognosis.^171^ To conclude, the prognostic significance of TLS varies according to tumor type, primary or recurrent, and so on. More precise grouping criteria and larger sample studies may help to further clarify the significance of TLS in serving as a prognostic marker.

### 4.2 TLS Regulation in immune therapy

Immunotherapy is a powerful clinical strategy in cancer treatment, which approximately encompasses ICB, ACT, and tumor vaccine strategies. Previous studies indicate that its therapeutic efficacy primarily influenced by TLS, immune checkpoint expression, TMB, and lymphocyte infiltration.^172^ Here, we will concentrate on clinical implications of modulating TLS during immunotherapy.

#### 4.2.1 Modulating TLS during ICB

Overall, employing inhibition of immune checkpoints is one of the most significant methods in immune therapy. Activating immune cells by blocking the “stop signal,” rather than just promoting their chemotaxis and expansion, can more effectively enhance their antitumor effect. ICB responders commonly have higher levels of antigen load and TILs with a higher proportion of effector cells while having fewer circulating MDSCs.^173^ TLS can also act as a signature of ICB responders. In 2021, Sweeney et al.^174^ reported intratumoral TLS activation and B‐cell expansion in a patient with sarcomatoid squamous‐cell carcinoma during treatment with the anti‐PD‐1 monoclonal antibody cemiplimab. Additionally, ICB therapy also holds promise in altering the protumor or antitumor phenotype influenced by TLS. Studies have reported high expression of immune checkpoint proteins in TLS structures within gastric cancer tissues, suggesting poor prognosis. ICB therapy may be more suitable for these patients.^175^

In order to alleviate resistance to ICB, targeting specific alternative molecular pathways holds significant therapeutic promise. These molecules are always associated with the function of certain cell subtypes, like Tregs (OX40 and 4‐1BB), NK cells (T cell immunoreceptor with immunoglobulin and ITIM domain, TIGIT), MΦ (TIM4), and T cells (TLR4). TNFRSF costimulatory molecules OX40 and 4‐1BB are constitutively expressed on Tregs, the major cellular components of inhibitory TLS. Melake et al.^17^ reported that OX40 was highly expressed on tumor‐infiltrating Tregs compared with Tregs in blood in UPS, myxofibrosarcoma, and DD liposarcoma. Furthermore, they found that TLS‐related Treg depletion could significantly increase the levels of CD4+T and CD8+T in TLS.^17^ Although T cells are the primary effector cells in ICB therapy, tumor‐resident NK cells also express checkpoint molecules like PD‐1, TIGIT, and CD96.^33^ Thus, focusing on NK cell surface molecules may uncover new targets. TIGIT is another intervention site to restore NK cell activity. In 2019, one research published in *Nature Immunology* reported that most NK cells express TIGIT and are exhausted in TIME, while NK cells expressing PD‐1 and CTLA4 only accounted for a small proportion, which limits the efficacy of applying ICB alone.^176^ To our interest, formation of TLS is a consequence of TIGIT blockade. Ozmadenci et al.^177^ reported that the combined application of focal adhesion kinase (FAK) inhibition and TIGIT blockade reduced TIGIT ligand CD155 levels, promoted TLS formation, and prolonged survival. TIM4 is a macrophage surface marker; however, the function of TIM4+MΦ varies greatly among cell subsets within and outside TLS. The enrichment of TIM4+MΦ in TLS was positively correlated with infiltration of immunocompetent cells and a better prognosis, whereas cavity‐resident TIM4+MΦ resulted in immunosuppression. Therefore, selective inhibition of TIM4 in non‐TLS tissues may be more effective.^72^

Poor immune infiltration, immunosuppressive cells, and specific extracellular matrix have been reported in osteosarcoma for the main cause of ICB resistance.^178^ Several attempts have been made to better activate immune cells within TLS in response to ICB treatment. Combination of toll‐like receptor (TLR) 4 activation and ICB has the potential to enhance the efficacy. In 2022, Italiano et al.^16^ used an intratumoral injection of TLR4 agonist (G100) combined with PD‐1 blockers (Pembrolizumab) and cyclophosphamide. TLR4 activation promoted lymphocyte infiltration but did not show obvious clinical benefits such as tumor shrinkage.^179^ It may be because Treg cells increase more than CD8+T cells, thus playing a dual role of protumor and antitumor.^180^ To further elucidate the factors influencing the response to ICB treatment, Italiano et al.^16^ analyzed the relative abundance of immune cells and the spatial expression of genes in TLS of nonresponders and responders. Genes of chemokines involved in TLS formation, such as CXCL12 and CCL18, were upregulated in responders, while nonresponder TLS showed significant upregulation of CTLA‐4 and marked enrichment of Tregs. The most striking difference was in the infiltration of plasma cells and their surface IgG expression, which is consistent with responsiveness to ICBs. These results suggest that the tumor‐specific antibody production and antigen presentation to activate CD8+T cells are the key events in TLS, which is linked to improved ICB efficacy and prolonged survival.^16^ These findings suggest that CTLA‐4 blockade or anti‐Treg therapy may be beneficial when added to conventional anti‐PD‐1 therapy. Another study from the same year combined a phosphoinositide 3‐kinase (PI3K) inhibitor with PD‐1 blockade. PI3K inhibitors reduced Treg activity, thereby promoting CD8+T expansion in TLS and enhancing ICB efficacy.^181^ In addition to this, promoting IgG+ plasma cells may also promote better activation of CD8+T cells. In addition to regulating the degree of infiltration and polarization of immune cells, targeting the regulation of the immunosuppressive microenvironment might strengthen the immune response. IDO1 has been reported to express in FDCs in TLS structure.^108^ Since it has the potential to inhibit CD8+ T cells, upregulate Tregs and polarize macrophages into immunotolerant phenotype,^163^ IDO1 targeting might enhance ICB response. Nevertheless, the combination blockade of IDO1 and PD‐1/PD‐L1 might be more appropriate for inflamed tumors, given that the driving factor for IDO1 expression in NSCLC patients is likely inflammation rather than the tumor itself.^108^ A Clinical Trial published in *JAMA Oncology* in 2018 showed that PD‐1 treatment of sarcomas, especially GIST, resulted in an immunosuppressive microenvironment characterized by macrophage infiltration and high expression of IDO1.^164^ In 2023, a study published in *Nature* reported that antibodies against endogenous retroviruses were shown to enhance B cell response and subsequent TLS formation in lung adenocarcinoma treated with ICB.^182^ Other therapies, like adoptive T cell transfer, DC vaccines, MDSCs inhibitors, oncolytic adenovirus, antiangiogenesis therapy, and radiotherapy, have shown their efficacy to activate immune response in TLS and enhance ICBs in osteosarcoma.^178^ To conclude, since TLS act as the site for ICB response, TLS‐specific immune activation can effectively improve ICB therapy.

#### 4.2.2 Modulating TLS in ACT therapy

In addition to ICB, ACT has also shown its promising activity.^183^ Low infiltration of immune cells in some tumors leads to ICB resistance but suggests a potential role for the ACT. ACT mainly includes TIL therapy, T cell receptor (TCR) therapy, and chimeric antigen receptor (CAR) therapy.^184^ TIL therapy refers to ex vivo expansion and reinfusion of TILs. As a combination of multiple lymphocytes, it has natural recognition of tumor cells. Lee et al.^185^ found that in BC tissues, the level of TILs and the number of TLSs jointly determine the level of ex vivo expansion. Even for samples with a low percentage of TILs, samples containing abundant TLSs yielded sufficient amplification.^185^ However, since TIL refers to nonspecific amplification, good expansion of TILs is not the only determinant of efficacy. The proportion of iconic memory phenotype T cells in TILs and whether the reactivity to the autologous tumor is preserved are also key parameters for predicting response.^185^ Another study compared pancreatic ductal adenocarcinoma (PDA) with melanoma, which was considered to respond less favorably to ACT. Although both of them had a diversity of T cell clones in tumor tissues and expanded well in vitro, PDA lost antigen‐specific T cells during in vitro expansion, thereby reducing the proportion of reactive T cells.^186^ Researchers also reported that TLS is enriched for antigen‐reactive T cells in PDA and positively correlates with the production of proinflammatory factors and better prognosis. Thus, increasing functional TLS, preserving the activity of its antigen‐specific T cells, and optimization of TILs culture are alternative strategies to improve ACT efficacy.^186^ In addition, researches showed that the liberation from immunosuppressive TIME is one cause of ex vivo expansion, especially the inhibitory effect of TGF‐β.^187^ This suggests that altering the inhibitory TME of TLS may help to improve the efficacy of TIL.

TCR or CAR therapy refers to the isolation of peripheral blood immune cells, mainly T cells, genetically modified to express specific antigen receptors, and then infused back into the patient. Stable and widely expressed tumor antigens remain to be discovered, especially those expressed in sarcomas. For example, cancer testis antigen, a common target for TCR therapy, is only stably expressed in certain sarcoma types, such as synovial sarcoma and myxoid/round cell liposarcomas, thus limiting its use.^172^ Another limitation of ACT therapy is its side effects. In CAR therapy,^188^ T cells are restricted to the autologous since allogeneic T cells can cause severe graft‐versus‐host disease (GvHD). However, NK cells do not cause GvHD, thus lifting this limitation. CAR‐NK has antitumor activity and a wider range of applications.^33^ It induced specific cytolysis of tumor cells in melanoma in the absence of sufficient immune cell infiltration, although its activity in vivo needs to be further confirmed.^189^ Memory B cells may also serve as an option for ACT. IgG1‐mutated B cells have been reported to have increased B cell receptor signaling and antitumor immunity.^190^ Ye et al.^191^ furtherly found that this leads to an increase in TLS density and area in the TIME. Therefore, the development of therapies that modulate BCR signaling can enhance the efficacy of adoptive transfer of memory B cells, thereby improving TLS reactivity. In conclusion, in addition to T cells, multiple components of TLS, including NK cells and B cells, have the potential to participate in ACT therapy. Changing the inhibitory microenvironment of TLS to increase the expansion of antigen‐specific lymphocytes is an alternative strategy.

Each cell type composing TILs is not isolated and has comprehensive crosstalk with each other. Thus, modulation of TIL has the potential to enhance ICB responses. Ozaniak et al.^192^ conducted a cohort study of patients after resection of soft tissue sarcomas, and found that a combination of anti‐PD‐1 and anti‐CD47 therapies showed a drastic reduction of proinflammatory cytokines. They speculate that they share similar cellular mechanisms, which results in competitive inhibition.^192^ While anti‐CD47 promotes phagocytosis of tumor cells by macrophages,^193^ it also promotes transformation to TAMs phenotype,^194^ which produces cytokines that inhibit CD8+ T cells and activate Tregs.^195^ Therefore, TIL‐based modulation is expected to enhance the effects of ICB.

#### 4.2.3 Modulating TLS in tumor vaccine

Tumor vaccine is another emerging immune therapy, including cell vaccine, protein or polypeptide vaccine, and nucleic acid vaccine. Previous research reported TLS formation and function during vaccine therapy. Combination therapy, such as protein vaccine combined with adjuvant,^196^ DC vaccine combined with dasatinib, increases the formation of TLS and enhances the immune response in melanoma.^197^ As a component of adjuvant, TLR3 agonist injection at the tumor vaccination site can promote the expansion of antigen‐specific T cells and enhance the immune response in TLS.^198^

### 4.3 HEV Induction in sarcoma treatment

HEV, a basic component of TLS, is a structurally and functionally specialized postcapillary vein. The endothelium expresses PNAd and CCL19/21, which interact with L‐selectin and CCR‐7 on T cells respectively, to mediate the entry of T cells into lymphoid tissues and participate in lymphocyte recycling.^199^ Previously, it was believed that HEV only occurred in SLOs, such as LNs and mucosa‐associated lymphoid tissue. However, recent studies have found that HEV markers can also be detected in tumor tissues, and correlate with a better prognosis of tumors.^200^, ^201^ The sign for HEV development is presumed to be the same as that required for LN organogenesis: LTα1β2 on LTi stimulates LTβR on stromal LTi cells to transmit signals for LN and HEV formation.^202^ naïve T cells specific for tumor antigens undergo priming and differentiation in situ once they are recruited through HEV, thus enhancing the antitumor effect.^203^ Furthermore, Zhan et al.^204^ evaluated the role of HEV in colorectal cancer and found that patients with higher HEV/TLS ratio had longer overall survival and disease‐free survival, and their TIME was more inclined to antitumor, that is, with more CD3+T, CD8+T, and M1 macrophages. Using a mouse model of metastatic melanoma, Asrir et al.^205^ confirmed that increasing the frequency and maturity of HEV promotes CD8+T cell infiltration and enhances the efficacy of anti‐PD‐1/anti‐CTLA combination therapy.

The promotion of HEV formation and other therapeutic methods promote each other. The vicinity of HEV is characterized by reduced angiogenesis, which is caused by the recruitment of immune cells.^206^ The combination of antiangiogenesis and anti‐PD‐1 therapy can also promote HEV formation.^207^ Prospective studies focused on TLS‐positive advanced cancer patients have further confirmed the potential of combined antiangiogenic therapy to overcome resistance observed with the sole use of ICB. A multikinase inhibitor is utilized to inhibit angiogenesis.^208^ Hua et al.^18^ demonstrated that the formation and maintenance of tumoral HEV requires a continuous supply of LTα1β2 by CD8+T cells and NK cells. Meanwhile, antivascular endothelial growth factor (VEGF) significantly enhanced HEV induction.^18^ Carbohydrate epitope MECA‐79 is primarily expressed on PNAd that are presented on HE. In endometrioid adenocarcinoma, higher MECA‐79 epitope levels on HEV ECs correlated with reduced tumor volume and parametrial infiltration.^209^ As for sarcoma, in 2021, Dridi et al.^210^ found low MECA‐79 levels in chordoma tissues using immunohistochemistry, which implies inhibited formation of HEVs and TLSs. Therefore, the induction of HEV in tumor tissue contributes to the increase of TLS levels. Of interest, Fleig et al.^211^ showed that loss of Notch signaling in vascular endothelium leads to conversion of arterial endothelium to HEV phenotype by conditional deletion of Notch signaling mediator RBPJ in mice. They also observed the spontaneous formation of TLS, indicating that HEV formation is mediated by the vascular endothelium rather than the lymphatic endothelium.^211^ To sum up, promoting HEV formation contributes to TLS genesis and its antitumor effect.

### 4.4 TLS induction by molecular drivers

The organization and maturation of TLS are modulated by a series of cytokines and chemokines, including LTα, LTβ, CXCL12/13, CCL19/21, IL‐7, IL‐17, IL‐22, IL‐23, and IL‐27.^212^ Immunohistochemistry showed that TLS chemokines, such as CXCL12 and CCL18, were upregulated in TLS‐positive patients who responded to Pembrolizumab combined with cyclophosphamide.^16^ Therefore, regulation of TLS‐related gene expression, or direct administration of chemokines, can promote the formation and maturation of TLS.

Since CXCL13 is involved in the formation of TLS in a series of malignant tumors, Groeneveld et al.^213^ measured CXCL13 transcript levels and suggested that CXCL13 could be used as a biomarker of TLS and correlated with prolonged survival and response to ICB. Similarly, Goubet et al.^214^ reported that CXCL13 was detected only in the plasma of responders and identified CXCL13‐producing Tfh as a preferential target for PD‐1 blockade. The accumulated Tfh and CXCL13 levels in tumor tissues are directly related to TLS in tumor tissues and can be used as biomarkers to predict progression‐free survival.^214^ A similar phenomenon has been reported in lung adenocarcinoma tissues.^215^ The sources of CXCL13 are different in the early establishment and later maintenance of TLS. Ukina et al.^216^ found that CXCL13 is mainly expressed by CD4+T cells mediated by TGF‐β in the early stage of TLS, and turns to CD21+FDC after TLS maturation, that is when FDC appears. However, the CXCL13/CXCR5 axis may act as a double‐edged sword. Bai et al.^217^ observed in hepatocellular carcinoma and BC that although applying Aspartate β‐hydroxylase‐targeted λ phage vaccine alone activates CXCL13/CXCR5 axis, it promotes an aggressive tumor phenotype and mediates effector T cell. Conversely, when PD‐1 blockade was combined with this λ phage vaccine, the CXCL13/CXCR5 axis contributes to TLS formation and promotes the cytotoxic effect of CTL on cancer cells. At the same time, they found that recombinant λ phage caused Th1 responses both in vitro and in vivo, effectively activated CD8+CTL responses, and produced a large number of antibodies. This suggests a significant enhancement of adaptive immunity by the combination of λ phage vaccine and ICB therapy.^217^

Other chemokines seem to interfere TLS occurrence and tumor inhibition. The activation of cyclic guanosine monophosphate‐adenosine monophosphate synthase (cGAS)‐stimulator of IFN genes (STING) pathway is a predictor of good response to cisplatin chemotherapy and PD‐1 blockade in lung cancer.^218^ This may be related to the function of cGAS as a sensor of pathogenic DNA,^219^ which has been entered into preclinical studies as an intervention target in hepatocellular carcinoma.^220^ Chelvanambi et al.^19^ applied low dose of STING agonist on a mouse melanoma subcutaneous tumor‐bearing model and found that tumoral TLS increased expression of antiangiogenic factors TNFSF15 and CXCL10 as well as TLS inducers such as CCL19/21, LTα/β, and LIGHT. Flow cytometry and immunofluorescence revealed enhanced CD8+T and CD11c+DC tumor infiltration. These effects all resulted in tumor vascular normalization, TLS formation, and controlled tumor growth.^19^ IL‐15 has been reported to activate STING pathway to promote TLS formation. He et al.^221^ demonstrated that oncolytic adenovirus encoding IL‐15 (Ad‐IL15) activated the STING‐TBK1‐IRF3 pathway in DCs, thereby promoting TLS formation and inhibiting tumor growth in a mouse model. In 2018, Hill et al.^222^ confirmed that the gp130/STAT3 pathway not only drives the occurrence of gastric adenocarcinoma, but also is directly related to the expression of CXCL13, CCL19, and CCL21, which initiate TLS genesis. Yin et al.^223^ found that tobacco exposure induced TLS formation by promoting CCL21 expression in epithelial cells, thereby improving immunotherapy efficacy, revealing the prognostic and therapeutic value of serum CCL21 levels.

Upstream of these TLS‐related chemokines also offer potential strategies. Of interest are LTs and IL‐7, which have been shown to sequentially participate in TLS formation. In 2022, Nayar et al.^224^ first reported that immunofibroblasts induced LTα3 production, which drove the expression of chemokines such as CCL19 and CXCL13. Pharmacological or genetic blocking of this pathway resulted in defects in TLS formation.^224^ IL‐7 is another TLS formation regulator which simultaneously participates in the remodeling of the lymphatic vascular network during the development of TLS. Nayar et al.^225^ confirmed that IL‐7 induced the early phase of LVs remodeling and LTα1β2 regulated the following formation of complex lymphatic networks. These upstream regulators are potential targets for future research.

In addition to activate relevant pathways, direct local injection is also a feasible strategy. Delvecchio et al.^226^ used a combination of systemic chemotherapy (gemcitabine) and local injection of lymphochemokine (CXCL13/CCL21) in an orthotopic PDA murine model to promote TLS formation. The antitumor activity, as indicated by reduced tumor volume and improved survival, was stronger than that of treatment alone. Immunofluorescence showed that this was dependent on local recruitment and enhanced interactions of immune cells in organized structures, especially B cell‐mediated DC activation.^226^

### 4.5 Other clinical implications for modulating TLS activity

In addition to increasing the number of TLS, improving their maturity also has potential therapeutic implications. Based on the fact that TGF‐β has the effect of downregulating the gene organizer SATB1, Chaurio et al.^227^ demonstrated that TGF‐β‐induced silencing of SATB1 promotes Tfh differentiation while inhibiting Treg production, thereby driving TLS maturation in ovarian tumors in situ and enhancing B‐cell responses. Therefore, inhibition of STAB activity by TGF‐β might enhance TLS function. Immunotoxin LMB‐100 also develops TLS to exert its antitumor activity.^20^ These effects were shown to require CXCL13‐dependent formation of TLS.^182^ Recently, the therapeutic significance of epigenetic modifiers has been reported in autoimmune diseases. Lysine demethylase 6B drives the acquisition of phenotype and function in immunofibroblasts, leading to the maturation of TLS.^228^ Other components of TIME, like neurons and vessels, also modulate TLS formation. Of interest is that tumor‐associated sensory neurons in melanoma would inhibit HEV maturation and T/B cell recruitment, thereby negatively regulating TLS formation.^229^ Recent findings also suggest that increased vascular density in intrahepatic cholangiocarcinoma promotes the occurrence of active immunity, leading to enhanced TLS formation.^230^ These finds supplest the potential for denervation or venogenesis therapy. It should also be noted that glucocorticoid treatment impairs the formation of germinal centers and thus inhibits the maturation of TLS.^51^ As observed in lung squamous cell carcinoma, the combined effects of chemotherapy and corticosteroid eliminated the prognostic value of TLS.^51^ In conclusion, elucidating the impact of existing treatment strategies on TLS or developing new therapies could help to enhance antitumor immune responses and improve efficacy.

### 4.6 Related side effects of TLS‐related treatments and prevention

TLS‐related treatments, especially immunotherapy, may be accompanied by side effects, mainly manifested as autoimmunity. The clinical application of immunotherapy is affected by immune‐related adverse events. Tsukamoto et al.^231^ reported age‐related adverse events with anti‐PD‐1 therapy. In aged mice, ICB leads to IL‐21 production by CD4+T, resulting in increased systemic CXCL13 levels.^231^ CXCL13 acts as a B cell homing chemokine, promoting B cell infiltration and IgG production in organs such as the lung, where IgG deposition leads to organ toxicity. Despite the antitumor role of CXCL13 in promoting TLS formation locally, CXCL13 was more frequently expressed in organs affected by side effects but not in tumor tissues in aged mice than in young mice. This may explain why high levels of circulating CXCL13 during ICB therapy do not indicate a better response. Therefore, targeting the IL‐21/CXCL13‐auto‐Ab axis is a possibility to circumvent adverse events of immunotherapy.^231^

## 5 CONCLUSIONS AND OUTLOOK

TLS, as an organized aggregate of lymphocytes, has been observed in a variety of malignant tumors. A series of retrospective cohort and preclinical studies have shown that TLS can predict immunotherapy responsiveness and suggest higher survival. Induction of TLS is the way to transform “cold tumors” into “hot tumors.” However, there are still a series of shortcomings in the current research, which requires further strengthening in the future. In addition, regarding the relationship between TLS and malignant tumors, the following issues are worth exploring in the future.

First, a clear definition of TLS and standardized methods for identification and quantification are currently lacking. How many lymphocyte aggregates can be defined as TLS, and whether some LA structures inconsistent with classical TLS can be identified as the early stage of TLS vary in different studies. Considering the copresence of B cells and FDCs, the use of CD20/CD23 colocalization as a marker of TLS is recommended.^232^ What is more, detection of TLS needs less invasive methods. The search for markers suggestive of TLS in body fluids and secretions is a possible direction. Besides, the use of information technology can detect and quantify the existence of TLS at multiple levels. Based on the genomic and transcriptome data of tumors, some scholars have used 12 chemokines associated with TLSs to identify and quantify them.^233^ Compared with manual assessment, automatic identification and quantification of TLS on pathological sections has higher specificity and sensitivity.^234^ The application of artificial intelligence holds great promise as a future direction,^64^ and deep learning algorithms have already been employed in the identification of TLS.^235^

Second, at present, the relationship between TLS and malignant tumors is mainly discussed in epithelial cancers, while there are few studies on mesenchymal sarcomas and nonsolid tumors. In addition, there is a lack of multicenter, large‐sample studies, and stronger evidence is needed to support the role of TLS in specific cancers.

Third, studies on the immune profile and TLS occurrence in cancer often focus on gene expression products such as marker molecules. Further research can explore the relationship between cancer driver genes and TLS formation from the genomic and transcriptome levels. Notably, peritumoral TLS and intratumoral TLS seem to have different functions, and the drivers of this differential localization remain unclear. In addition, the mechanisms by which these two TLS affect tumor immunity need to be further elucidated. Future studies should focus not only on the presence and composition of TLS, but also on its localization in tissues.

Finally, the current therapeutic significance of TLS focuses on its use as a biomarker of efficacy and prognosis. Many immunotherapies can promote the formation of TLS. However, whether directly inducing TLS formation by biological or chemical means contributes to antitumor immunity is still poorly studied, and clinical trials need to be promoted.

In conclusion, for highly heterogeneous cancers, clarifying the formation mechanism and function of TLS in specific tumor types could facilitate the development of more effective therapeutic strategies, thereby enhancing the immune reactivity of cancers to improve efficacy and survival.

## AUTHOR CONTRIBUTIONS

C. T. conceived and designed the work. S. Y. W. and H. W. wrote the paper. C. B. L. and B. F. L. collected and analyzed the relevant reports. C. T. and S. S. H. provided substantial contributions to improve the content of the article. All authors have read and approved the manuscript.

## CONFLICT OF INTEREST STATEMENT

The authors declare no conflict of interest.

## ETHICS STATEMENT

Not applicable.

## Data Availability

Not applicable.
